# Chloroprocaine 3% Gel as a Novel Ocular Topical Anesthetic: Results from a Multicenter, Randomized Clinical Trial in Patients Undergoing Cataract Surgery

**DOI:** 10.1089/jop.2023.0096

**Published:** 2024-03-14

**Authors:** Michele Figus, Fabrizio Giansanti, Edoardo Villani, Jorge L. Alió, Ladislav Jančo, Stefano Mercuri, Stefano Camnasio, Carlo Cagini

**Affiliations:** ^1^ Department of Surgical, Medical, Molecular Pathology and Critical Care Medicine, University of Pisa, Pisa, Italy.; ^2^ Department of Neurosciences, Psychology, Drug Research and Child Health, University of Florence, Florence, Italy.; ^3^ Eye Clinica, San Giuseppe Hospital, IRCCS Multimedica Milano, Milan, Italy.; ^4^ Opthalmology Department, Vissum Miranza Alicante, Alicante, Spain.; ^5^ Očná klinika SZU Nám., F.D.Roosevelt Hospital, Banská Bystrica, Slovakia.; ^6^ Scientific Department, Sintetica SA, Mendrisio, Switzerland.; ^7^ Department of Medicine and Surgery, Section of Ophthalmology, University of Perugia, Perugia, Italy.

**Keywords:** chloroprocaine, cataract surgery, topical ocular anesthesia, ocular procedure

## Abstract

**Purpose:**

To compare the efficacy and safety of a novel ophthalmic anesthetic, chloroprocaine 3% gel to tetracaine 0.5% eye drops in patients undergoing cataract surgery with phacoemulsification.

**Methods:**

This was a prospective, randomized, multicenter, active-controlled, masked-observer, parallel group competitive equivalence study. The study comprised 338 patients having routine cataract extraction by clear corneal phacoemulsification, randomized to receive 3 drops of chloroprocaine gel (*n* = 166) or tetracaine eye drops (*n* = 172) before surgery. The primary objective of the study was to assess the equivalence of chloroprocaine gel to tetracaine eye drops as proportion of patients with successful ocular surface anesthesia, without any supplementation just before intraocular lens implantation. Safety measurements were pain, irritation, burning, stinging, photophobia, and foreign body sensation, graded by the patient and objective ocular signs.

**Results:**

Equivalence was demonstrated, with a somewhat higher success rate of chloroprocaine gel: 152/166 (92.0%) chloroprocaine versus 153/172 (90.5%) tetracaine patients achieved ocular surface anesthesia with no supplementation. Difference in proportions was 1.5% confidence interval [95% CI: (−3.6 to 6.6)] and 90% CI fell within (−10 to 10). Mean onset of anesthesia was 1.35 ± 0.87 min for chloroprocaine and 1.57 ± 1.85 for tetracaine (*P* = 0.083). Mean duration of anesthesia was 21.57 ± 12.26 min for chloroprocaine and 22.04 ± 12.58 for tetracaine (*P* = 0.574). No treatment emergent adverse events related to chloroprocaine were reported and no relevant findings related to local tolerance or vital signs were observed in both arms.

**Conclusions:**

Results obtained from the present cataract study demonstrated that chloroprocaine 3% ophthalmic gel is safe and effective, representing a valid alternative in ocular topical anesthesia.

Clinical Trial Registration number: NCT04685538.

## Introduction

Topical local anesthetics (LAs) play an important role in the practice of ophthalmology, especially in noninvasive surgeries. Continuously improving surgical techniques require less extensive anesthesia and decrease the need for hospitalization and postoperative controls. As a matter of fact, many ophthalmic procedures, including cataract surgery, intravitreal injections, and minor diagnostic procedures, are at present commonly performed using topical LA agents along with other agents.

Cataract surgery is the most frequent surgical procedure performed in the world, with a yearly rate approaching 1% of the entire population in Western countries.^1^ The procedure lasts less than 15 min in uncomplicated cases and is performed on an outpatient basis. Success rate is high and intraoperative or postoperative complications are very rare. As a result, the retrobulbar or peribulbar needle anesthesia that had been used for more than a century has been almost entirely abandoned, remaining an option only when a certain degree of akinesia is desired or when the surgery is particularly complicated to require a longer duration of the anesthetic effect.^2^

Nowadays topical anesthesia represents the first choice in cataract surgery because there is no blood in cut or touched tissues that could remove the anesthetic drug, eyeball akinesia is no longer required, additional anesthesia can be applied at any time, and there is no risk of globe perforation by needles.^3,4^ LAs are topically applied to the eye and act directly on the corneal epithelium and stroma. The portion of drug penetrating into the anterior chamber suppresses pain arising from the iris and ciliary body by reversibly blocking voltage-gated sodium channels. Sensory termination block is the most important feature of topical anesthesia. It involves the inhibition of sodium channels at nerve endings or receptors by the anesthetic agents, thus blocking the production (and not the transmission) of nervous impulses.^2^

First approved ocular topical anesthetic products included solutions such as oxybuprocaine, proparacaine, or tetracaine drops. Subsequently, reports on off-label use of lidocaine 2% gel for ophthalmic procedures showed a favorable if not better profile which led to the development of lidocaine 3.5% [approved by the U.S. Food and Drug Administration (FDA) in 2008] and lidocaine 2% (approved by EMA in 2021) ophthalmic gel products indicated for topical anesthesia during ophthalmic procedures.^5,6^

Indeed, the viscosity of gel formulations increases residency time on the ocular surface of the LA which results in increased drug exposure in the deeper tissues and reduces its systemic absorption, following topical administration. The studied chloroprocaine ophthalmic gel 3% has a viscosity not exceeding 2,000 cps compared to other FDA approved topical anesthetic gel agents such as Akten's (lidocaine ophthalmic gel 3.5%) 4,000–9,000 cps and lidocaine jelly's 12,000–14,000 cps. On the contrary, topical anesthetic solutions with lower viscosity, such a tetracaine 0.5% ophthalmic solution with a viscosity between 15 and 25 cps, are rapidly cleared from the surface of the cornea through the nasolacrimal drainage system and systemically absorbed through the nasal mucosa.^5,7–10^

This study evaluates the efficacy and safety of chloroprocaine ophthalmic gel 3%, a new preservative-free, single-use ophthalmic preparation, in patients undergoing cataract surgery with phacoemulsification compared to tetracaine ophthalmic solution 0.5%, a proven and trusted standard for ophthalmic anesthesia during cataract surgery.

Favorable results generated from this study contributed to chloroprocaine 3% ophthalmic gel's recent approval by the FDA (2022) under the brand name IHEEZO™ (Harrow, Inc., Nashville, TN).

Chloroprocaine, first synthesized in 1946 from procaine, is a short-acting LA belonging to the amino ester class, characterized by a rapid onset of action (usually 6 to 12 min) and a duration of up to 60 min, depending on the dose and the route of administration.^11^ Due to its rapid hydrolysis by pseudocholinesterase, the systemic toxicity of chloroprocaine is virtually nonexistent and, therefore, chloroprocaine is widely considered the LA with the safest toxicological profile.

Expected benefits of chloroprocaine, here assessed for the first time to provide ocular surface anesthesia as a topical gel formulation, include the following: coating of the eye without requiring repeated doses (repeated use of topical ophthalmic anesthetic either in frequency of application or length of time of use, can result in serious ocular complications^12^); tendency to stay on the eye for a longer duration than liquid formulations without being absorbed by the lacrimal puncta as quickly as the tears; reduced systemic absorption through the nasolacrimal system which translates into reduced potential for systemic toxicity; improved lubrication of surgical instruments with easier entry and exit through surgical wounds.

## Methods

This was a prospective, randomized, multicenter, active-controlled, masked-observer, parallel group competitive phase III equivalence study in outpatient surgery centers.

The trial was conducted between September 2020 and March 2021 at 20 centers in 3 countries (Italy, Slovakia, Spain) after obtaining IRB approval and in accordance with the relevant guidelines of the Declaration of Helsinki and the International Conference on Harmonization guidelines on Good Clinical Practice (ICH E6). Signed informed consent was obtained from all patients. The study was registered with EudraCT number 2019-001660-30 and Clinicaltrials.gov identifier.

The primary objective of the study was to assess the equivalence of chloroprocaine 3% gel versus tetracaine 0.5% eye drop in terms of proportion of patients with successful ocular surface anesthesia, without any supplementation, just before intraocular lens implantation (T4).

The secondary study objectives were to compare the clinical efficacy and safety of chloroprocaine 3% gel to those of tetracaine 0.5% eye drop.

The study included a Selection/Baseline visit (Visit 1—Day-90/Day-1), an Inclusion visit (Visit 2—Day 1/surgery day), a Follow-up visit (Visit 3—Day 2, phone call), a Final visit (Visit 4—Day 8), and an Optional visit in case of adverse events (AE) at visit 4 (Visit 5—Day 28, phone call).

On the day of surgery, patients were randomized 1:1 to receive either chloroprocaine 3% gel or tetracaine 0.5% eye-drop solution (Bausch&Lomb UK Ltd.), according to a computer-generated randomization list. For both treatments, 3 drops were instilled as follows: first drop instillation, wait for 5 min, eye disinfection, wait for 2 min, second drop instillation, wait for 1 min, third drop instillation, and wait for 1 min, start of surgery.

In this study, the surgeon who instilled either chloroprocaine 3% gel or tetracaine 0.5% eye drop was aware of the treatment administered and, apart from cataract surgery, was only involved in patient surgery and in surgeon satisfaction assessment. All study variables, collected and analyzed for primary and secondary objectives were observed, recorded, and clinically evaluated by another independent investigator, masked to the formulation applied. The masked investigator performed screening assessments and assessed primary endpoint at Visit-1/Selection (patient discomfort during surgery), then patient global satisfaction and AEs occurrence at Visit-3/Follow-Up, and overall secondary endpoints (clinical efficacy and ocular and systemic safety parameters) for each patient at Visit-4/Final. Patients were also masked to the administered product.

Surface anesthesia success was assessed at time before first incision (T1), end of capsulorhexis (T2), end of phacoemulsification (T3), and just before intraocular lens implantation (T4) time points during surgery using a multiple-point ordinal scale:

- 0—“no pain or discomfort”;
- 1—“occasional pressure sensation”;
- 2—“occasional burning or stinging sensation, less than 5 separated times during procedure”;
- 3—“occasional burning or stinging sensation, more than 5 separated times during procedure”;
- 4—“continuous sensation of stinging, burning, or pressure during procedure, tolerable”;
- 5—“sensations in point 3 intensified, described as severe or nontolerable.”

Based on the scale, successful surface anesthesia was defined when no pain/discomfort (scale = 0) or occasional pressure sensation, less than 5 separated times during procedure (scale = 1) were reported by the patient. Supplementation was defined as any intraoperative analgesia, including local anesthesia after the beginning of the surgery. Mild sedation before the start of the surgery was allowed for anxious patients, according to surgeon experience, and could be used during surgery in situations where anxiety and/or movement of the patient could prevent the surgeon from performing the procedure in a sufficiently safe manner. In such cases, patients were excluded from the per-protocol analysis but were not considered as failure.

The secondary objectives of the study included patient's discomfort assessment during surgery at T1, T2, and T3, blink reflex at the end of surgery, time to achieve anesthesia, total anesthesia time, total surgical time, surgeon assessment, and safety measures, including endothelial cell counts, corneal thickness, best corrected visual acuity, fundoscopy, corneal fluorescein staining, intraocular pressure (IOP), and vital signs [blood pressure (BP)] and heart rate.

Time to obtain sufficient anesthesia (onset of anesthesia) was defined as the time between the last drop of anesthesia given and the time anesthesia is confirmed with forceps, just before start of surgery, while duration of anesthesia (total anesthesia time) was defined as the interval between the time to obtain sufficient anesthesia (assessed by the surgeon with forceps after the third drop instillation, just before start of surgery) and the end of anesthesia (assessed with forceps every 5 min from the end of surgical intervention). To assess the duration of anesthesia, the testing was considered as concluded when the subject reported pain on 2 successive tests with 5 min in between; time of the first test was then formally considered as the end of anesthesia.

Patients who presented intraoperative complications preventing primary endpoint assessment were withdrawn from the efficacy analysis and the standard treatment of the hospital was to be applied.

Surgical comfort was assessed using the following scale: 0—“uncomplicated”; 1—“slightly complicated”; and 2—“complicated.”

Patient global satisfaction at Follow-up visit (Visit 3—D2) was assessed using the following scale: “Very satisfied”; “Globally satisfied”; “Neither satisfied nor unsatisfied Globally”; “Unsatisfied” and “Very unsatisfied.”

### Statistical analysis

In equivalence studies, sample size depends on the type I error (α) (which is usually set equal to 0.05), on the type II error (β) (set equal to 0.20 for the present trial, corresponding to a power of 80%), the equivalence margin *d* and the expected proportion of success in both treatment groups.^13^ For the expected equivalence *d* = 0.10, which corresponds to a difference in efficiency of 20%, the estimated sample size was 171 patients per group, after adjusting for a possible exclusion rate of 10%. For this reason, 171 patients per arm, that is, 342 patients overall, were to be enrolled.

Data and parameters measured were evaluated and presented using descriptive statistics, that is, arithmetic mean, standard deviation, minimum, median, and maximum values for quantitative variables, and absolute and relative (%) frequencies for qualitative variables. Statistics was reported by treatment group, separately for the eye to be operated and for the other eye. The proportion of patients with successful anesthesia without any supplementation at T1, T2, T3, and T4 (T4: primary efficacy endpoint) was evaluated in the operated eye using the Mantel-Haenszel approach (adjusting for country using random effects) to assess equivalence of chloroprocaine 3% with the reference product.

In terms of statistical inference for the remaining secondary endpoints, quantitative variables were compared between the 2 groups using Mann–Whitney's test. Qualitative variables, such as the assessment of patient's discomfort (T1–T4), use of supplementary treatments necessary for obtaining and/or maintaining anesthesia, and the occurrence of AE, were compared using Pearson's χ^2^ test. Time to obtain sufficient anesthesia and total time were calculated using mixed effects models (adjusting for center and country random effects).

Confidence intervals (CIs) were reported, where appropriate. These intervals were 2-sided in each case and provide 95% confidence.

The following analysis sets were considered: (1) Enrolled Set: all patients enrolled in the study. (2) Safety Set: all patients enrolled in the study, for whom there was evidence that they used study medication and for whom any follow-up information was available. (3) Full Analysis Set (FAS): all patients enrolled in the study for whom any follow-up efficacy information was available. (4) Per Protocol Set (PPS): all patients of the FAS who did not show any major protocol violation.

The statistical analyses of all secondary endpoints were conducted on the PPS and confirmed on the FAS. The primary population for the assessment of efficacy was the PPS, while the statistical analysis on the FAS population was considered as sensitivity analysis.

Imputation methods were envisaged for the replacement of missing values in each set only for the primary endpoint (anesthesia success).

In terms of AE, analysis was performed by the treatment group using descriptive statistics.

## Results

**FIG. 1.**
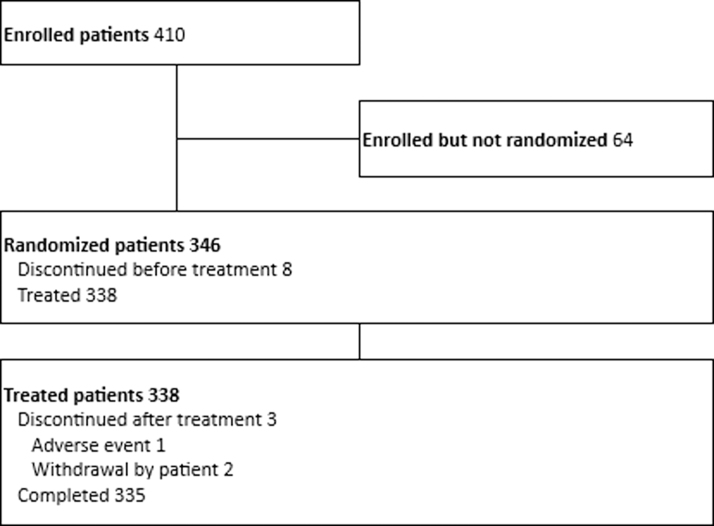
Participant flow figure.

In total, 410 subjects were enrolled in the study, 64 of which were dropped as screen failure and 346 were randomized to receive treatment. Of the 346 subjects randomized, 338 were randomized and treated according to the randomization procedure, while 8 discontinued before treatment. Eventually, 335 subjects completed the study (163 chloroprocaine, 169 tetracaine) since discontinuation issues occurred for 3 subjects: 1 in the tetracaine group due to AE and 2 upon the patients' wish to withdraw from the study (Fig. 1).

No imputation method for the replacement of missing values was applied during the analysis since none was observed in the collected data.

The average age of patients enrolled in all sets was 69 years, 53% were female and 47% male. All female patients had a reliable birth control method and none of them was lactating (Table 1).

**Table 1.** Demographics—Enrolled Set, Safety Set, Full Analysis Set, and Per Protocol Set

		Enrolled set	Safety set	Full analysis set	Per protocol set
Sex					
Female	*N* (%)	228 (55.6)	180 (53.3)	180 (53.3)	176 (53.0)
Male	*N* (%)	182 (44.4)	158 (46.7)	158 (46.7)	156 (47.0)
Age					
	*N*	410	338	338	332
	Mean	69.37	69.58	69.58	69.56
	Median	71.00	71.00	71.00	71.00
	SD	10.11	10.12	10.12	10.19
	Min	37	37	37	37
	Max	92	92	92	92
Birth control method					
Yes	*N* (%)	6 (2.6)	5 (2.8)	5 (2.8)	5 (2.8)
Childbearing potential					
No	*N* (%)	222 (97.4)	175 (97.2)	175 (97.2)	171 (97.2)
Yes	*N* (%)	6 (2.6)	5 (2.8)	5 (2.8)	5 (2.8)
Lactating					
No	*N* (%)	6 (2.6)	5 (2.8)	5 (2.8)	5 (2.8)

The number and the proportion of patients of each sex are reported.

The denominator for calculation of the proportions.

SD, standard deviation

### Efficacy

At T4 (just before intraocular lens implantation) 150/163 patients (92.0%) in the chloroprocaine group and 153/169 patients (90.5%) in the tetracaine group achieved surface anesthesia with no supplementation. The difference in proportions was 1.5% (95% CI: [−3.6 to 6.6]) and the associated 90% CI falling within the predefined equivalence interval (−10 to 10) (Table 2). Thus, clinical equivalence between chloroprocaine 3% gel and tetracaine 0.5% eye drop was established.

**Table 2.** Successful Surface Anesthesia Proportion at T4 in Visit 2 (Per-Protocol Set)

	Anesthesia success	Chloroprocaine 3% gel* n *(%)	Tetracaine 0.5% eye drops* n *(%)	Difference in proportions (%)*^a^*	90% 2-sided CI on the difference in proportions (%)	Rejection of null hypothesis*^b^*
Successful surface anesthesia^c^	Yes	150 (92.0)	153 (90.5)	1.5	(−3.6 to 6.6)	Yes
	No	13 (8.0)	16 (9.5)			

Patients are summarized according to the product they actually received.

^a^ Mantel-Haenszel approach for estimation of the common risk difference adjusting for country effect.

^b^ The null hypothesis states that the difference in proportion between the 2 treatment arms is outside the interval (−10%, 10%). If null hypothesis is rejected equivalence between the 2 treatment arms is established.

^c^ Successful surface anesthesia is defined as no pain or discomfort or ocular pressure sensation, less than 5 separated times during procedure without any supplementation at T4.

CI, confidence interval.

Similarly, no statistically significant difference between the 2 treatments was observed in the percentage of patients achieving surface anesthesia at T1, T2, and T3. In chloroprocaine 3% gel group, the percentage of patients who achieved surface anesthesia was 95% at T1, 97.6% at T2 and 93.3% at T3 versus 96.4% at T1 and T2 and 89.3% at T3 in the tetracaine 0.5% group. At all time points, the difference in the estimated proportions of success was less than 4% and the 90% CI fell within the predefined equivalence interval (−10 to 10) (Table 3).

**Table 3.** Successful Surface Anesthesia at T1, T2, and T3 in Visit 2 (Per-Protocol Set)

	Time point	Anesthesia success	Chloroprocaine 3% gel *n* (%)	Tetracaine 0.5% eye drops *n* (%)	Difference in proportions (%)^a^	90% 2-sided CI on the difference in proportions (%)	Rejection of null hypothesis^b^
Successful surface anesthesia^c^	T1—Just before first incision	Yes	155 (95.1)	163 (96.4)	−1.4	(−5.4 to 2.4)	Yes
		No	8 (4.9)	6 (3.6)			
	T2—End of capsulorhexis	Yes	159 (97.5)	163 (96.4)	1.0	(−2.0 to 4.1)	Yes
		No	4 (2.5)	6 (3.6)			
	T3—End of phacoemulsification	Yes	152 (93.3)	151 (89.3)	3.8	(−1.2 to 8.9)	Yes
		No	11 (6.7)	18 (10.7)			

Patients are summarized according to the product they actually received.

^a^ Mantel-Haenszel approach for estimation of the common risk difference adjusting for country effect. Due to numerical issues in the calculation of the common risk difference in T1, analysis was performed on the overall data (no country effect).

^b^ The null hypothesis states that the difference in proportion between the 2 treatment arms is outside the interval (−10% to 10%). If null hypothesis is rejected, equivalence between the 2 treatment arms is established.

^c^ Successful surface anesthesia is defined as no pain or discomfort or ocular pressure sensation, less than 5 separated times during procedure at T1, T2, and T3.

For both treatment groups median time to obtain surface anesthesia and mean duration of anesthesia was 1 and 22 min, respectively.

None of the patients in the chloroprocaine group required supplemental therapy to manage pain compared to 2 patients in the tetracaine group.

No statistically significant difference was found in surgical comfort. The mean values in both groups at all time points did not exceed 0.1 implying that the number of slight/severe complications at the different stages of the surgery was limited in both groups.

No significant difference between the 2 treatment groups in the overall patient satisfaction concerning the topical product was observed at Visit 3-Follow-up.

### Safety

In this study, the number of treatment emergent adverse events (TEAEs) was higher in the tetracaine group (26 events in 19 patients) than in the chloroprocaine group (16 events in 14 patients) (Table 4). Eye disorders accounted for 10 events in 10 patients in the chloroprocaine group and for 13 events in 10 patients in the tetracaine group. Corneal edema (5 events in 5 patients) of mild and moderate level was the most frequently observed TEAE in the chloroprocaine group. Increased BP (3 events in 3 patients) and increased IOP (6 events in 3 patients) were the most frequently observed TEAEs in the tetracaine group. None of TEAE was severe (10 mild and 6 moderate).

**Table 4.** Patients with Treatment-Emergent Adverse Events by System Organ Class and Preferred Term—Safety Set

System organ class	Chloroprocaine 3% gel	Tetracaine 0.5% eye drops
	*N* = 166	*N* = 172
	*n *(%) (nAE)	*n *(%) (nAE)
TEAEs	14 (8.4) (16)	19 (11.1) (26)
Congenital, familial and genetic disorders	0 (0.0) (0)	1 (0.6) (1)
Corneal dystrophy	0 (0.0) (0)	1 (0.6) (1)
Eye disorders	10 (6.0) (10)	12 (7.0) (13)
Conjunctival hemorrhage	1 (0.6) (1)	0 (0.0) (0)
Conjunctivitis allergic	0 (0.0) (0)	1 (0.6) (1)
Corneal degeneration	0 (0.0) (0)	1 (0.6) (1)
Corneal disorder	0 (0.0) (0)	1 (0.6) (1)
Corneal edema	5 (3.0) (5)	2 (1.2) (2)
Eye discharge	0 (0.0) (0)	1 (0.6) (1)
Hyperesthesia eye	1 (0.6) (1)	0 (0.0) (0)
Iridocele	0 (0.0) (0)	1 (0.6) (1)
Lens dislocation	0 (0.0) (0)	1 (0.6) (1)
Ocular hypertension	0 (0.0) (0)	1 (0.6) (1)
Photophobia	1 (0.6) (1)	1 (0.6) (1)
Punctate keratitis	1 (0.6) (1)	1 (0.6) (1)
Pupillary deformity	0 (0.0) (0)	1 (0.6) (1)
Pupillary disorder	0 (0.0) (0)	1 (0.6) (1)
Retinal pigment epitheliopathy	1 (0.6) (1)	0 (0.0) (0)
General disorders and administration site conditions	2 (1.2) (2)	0 (0.0) (0)
Pyrexia	1 (0.6) (1)	0 (0.0) (0)
Sensation of foreign body	1 (0.6) (1)	0 (0.0) (0)
Injury, poisoning, and procedural complications	1 (0.6) (1)	1 (0.6) (1)
Incision site edema	1 (0.6) (1)	0 (0.0) (0)
Procedural pain	0 (0.0) (0)	1 (0.6) (1)
Investigations	1 (0.6) (1)	6 (3.5) (9)
Blood pressure increased	0 (0.0) (0)	3 (1.7) (3)
Intraocular pressure increased	1 (0.6) (1)	3 (1.7) (6)
Nervous system disorders	0 (0.0) (0)	1 (0.6) (1)
Trigeminal neuralgia	0 (0.0) (0)	1 (0.6) (1)
Product issues	1 (0.6) (1)	1 (0.6) (1)
Device dislocation	1 (0.6) (1)	1 (0.6) (1)
Respiratory, thoracic and mediastinal disorders	1 (0.6) (1)	0 (0.0) (0)
Cough	1 (0.6) (1)	0 (0.0) (0)

Patients are summarized according to the product they actually received.

The number and the proportion of patients with any adverse event and the number of adverse events for each classification level are reported.

The denominator for calculating the proportions is the number of patients in the safety set of each treatment group.

System Organ Class and Preferred Terms are coded using MedDRA version 23.0.

TEAEs, treatment emergent adverse events.

None of 14 TEAEs reported in the chloroprocaine group was judged to be treatment-related, whereas 2 out of 19 TEAEs observed in the tetracaine group were judged to be treatment-related (corneal disorder and iridocele) (Tables 5 and 6).

**Table 5.** Global Incidence of Patients with Treatment-Emergent Adverse Events and Serious Treatment-Emergent Adverse Events—Safety Set

	Chloroprocaine 3% gel* N* = 166* n *(%)* (nAE)*	Tetracaine 0.5% eye drops* N* = 172* n *(%)* (nAE)*
TEAEs	14 (8.4) (16)	19 (11.0) (26)
Relationship to the drug		
Related	0 (0.0) (0)	2 (1.2) (2)
Not related	14 (8.4) (16)	17 (9.9) (24)
Severity		
Mild	10 (6.0) (10)	13 (7.6) (16)
Moderate	5 (3.0) (6)	9 (5.2) (10)
Severe	0 (0.0) (0)	0 (0.0) (0)
Serious TEAEs	0 (0.0) (0)	0 (0.0) (0)
Leading to discontinuation	0 (0.0) (0)	1 (0.6) (1)

Patients are summarized according to the product they actually received.

Patients are summarized according to each level of relationship and severity reported in each treatment group.

The number and the proportion of patients with any adverse event and the number of adverse events for each classification level are reported.

The number and the proportion of patients in each classification do not sum up to the total due to patients with multiple adverse events across the different classification levels.

The denominator for calculating the proportions is the number of patients in the safety set of each treatment group.

**Table 6.** Patients with Treatment-Emergent Adverse Events Related to the IMP by System Organ Class and Preferred Term—Safety Set

System organ class*^a^*	Chloroprocaine 3% Gel	Tetracaine 0.5% eye-drop
	*N* = 167* n *(%)* (nAE)*	*N* = 171* n *(%)* (nAE)*
TEAEs	0 (0.0) (0)	2 (1.2) (2)
Eye disorders	0 (0.0) (0)	2 (1.2) (2)
Corneal disorder	0 (0.0) (0)	1 (0.6) (1)
Iridocele	0 (0.0) (0)	1 (0.6) (1)

Patients are summarized according to the product they actually received.

The number and the proportion of patients with any adverse event and the number of adverse events for each classification level are reported.

The number and the proportion of patients in each classification do not sum up to the total due to patients with multiple adverse events across the different classification levels.

The denominator for calculating the proportions is the number of patients in the safety set of each treatment group.

^a^ System organ class and preferred terms are coded using MedDRA version 23.0.

In relationship to other secondary endpoints concerning local tolerability, no statistically significant differences for any of the ocular symptoms (foreign body sensation, irritation/burning/stinging, pain, and photophobia) were observed between the treatment groups, neither at Visit 1-Screening nor at Visit 4-Final. During slit lamp examination, no significant difference between the 2 groups was observed for the corresponding parameters. Corneal fluorescein staining showed no statistically significant difference between chloroprocaine and tetracaine for any eye. No statistically significant differences between treatment groups, at any time point, were observed for fundoscopy. There were also no significant differences in the mean IOP between the 2 groups at Visit 1-Selection and at Visit 4-Final. Optic coherence tomography showed no significant difference for retinal thickness between treatment groups and observed eyes at Visit 1-Selection.

## Discussion

The purpose of this study was to compare the efficacy and safety of a novel ophthalmic anesthetic, chloroprocaine 3% gel, to tetracaine 0.5% eye drops in patients undergoing cataract surgery with phacoemulsification.

Although an ideal comparator may have been a topical anesthetic in a gel formulation, at the time the study was conducted, no approved gel formulations were available in Europe. Therefore, 0.5% tetracaine eye drop solution was chosen as the most representative topical anesthetic used worldwide in patients undergoing cataract surgery with phacoemulsification.^7,13–17^

Chloroprocaine and tetracaine are very well known and clinically useful procaine-like LAs. They are amino esters, readily hydrolyzed by plasma esterases. It is now generally accepted that the mechanism of action of these drugs is based on the interaction with specific binding sites within the Na^+^ channels resulting in the blockade of the Na^+^ current.^18^ Both drugs block the generation and conduction of nerve impulses by increasing the threshold for electrical excitation in the nerve, by slowing the conduction of nerve impulses, and by reducing the rate of rise of the action potential.

The most important clinical properties of topical LAs are (1) potency, (2) onset, (3) duration of action, and (4) blockade of sensory fibers. Such qualities are primarily dependent on their physicochemical properties. Their potency and duration of action depend mainly on their lipid solubility, protein-binding and pKa: in general, lipid solubility determines the relative intrinsic potency of an anesthetic, protein binding influences the duration of anesthesia, while the pKa is correlated with the onset of action.^19^

The most common route of administration of ophthalmic drugs is the topical route because it is convenient, noninvasive, and accessible to all patients. Eye drops of aqueous solution are the preferred method of drug administration due to their easy handling, convenience, and relatively low cost. Unfortunately, their use is strongly limited by their poor ocular bioavailability (<5%)^20^ and the need to include preservatives that have demonstrated toxicity on the cornea. Therefore, to obtain a therapeutic efficacy, it is essential to reach higher concentrations in the ocular tissues. This can cause side effects such as toxicity and low tolerability.

The results of this phase 3 study showed that the new chloroprocaine 3% gel, here assessed for the first time to provide ocular surface anesthesia, might play an important role in the practice of ophthalmology, especially for routine ocular procedures.

Cataract surgery represents the most common medical condition that needs to be treated by means of outpatient surgery in ophthalmology and, therefore, it is considered highly representative for the future clinical use of chloroprocaine 3% gel. Global prevalence of cataract in adults over 50 years of age was estimated at 47.8%.^21^ The prevalence of cataract in Europe increased with age from 5% for the 52–62 years age group and 30% for 60–69 years of age to 64% for the population older than 70 years.^22^ There is currently limited literature evaluating the efficacy of topical local anesthesia alone during cataract surgery. One study evaluated the use of topical lidocaine 2% only during cataract surgery and found that 35% (37/106) first-eye cataract surgeries reported intraoperative pain. This number increased in second eye patients with 87% (46/52) reporting pain.^23^

Chloroprocaine 3% gel administration demonstrated highly successful and reliable ocular surface anesthesia, with rapid onset (1 min), a duration of anesthesia around 22 min. Specifically, 150 out of 163 patients (92.0%) achieved a successful surface anesthesia, without any supplementation just before intraocular lens implantation. In addition, no patients in the chloroprocaine 3% gel arm were administered with supplemental treatment for obtaining or maintaining anesthesia.

Current practices in North America suggest that it is not uncommon to use a preoperative or intraoperative dose of an opioid to keep patients comfortable and prevent intraoperative pain. Such a supplementation, however, may trigger postsurgical recovery complications. Approximately 20,000 prescriptions for postoperative opioids are administered annually following cataract surgery.^24^ Interestingly, a Mayo Clinic study analyzed sedation and recovery of 20,116 ophthalmic surgeries, 76.1% being cataract surgeries. Overall, 79.5% of those ophthalmic procedures received IV fentanyl as part of their anesthesia protocol.

The study found that patients who received IV fentanyl had a prolonged recovery time compared to patients who did not. Furthermore, patients with prolonged recovery had higher rates of emergency department visits and hospitalization in the first 48 h postop and higher 30-day mortality rates.^25^ Another retrospective analysis of 3,764 routine cataract surgeries performed by 33 surgeons at Duke University found that 97% of cases received IV fentanyl during surgery on top of topical tetracaine 0.5% solution.^26^ Zakrewski et al., evaluated 1957 routine cataract surgeries in Canada and found that 83.1% of patients received fentanyl for the procedure.^27^

Of note, no patients in this study received any pre- or intraoperative opioids. Importantly, no patient treated with chloroprocaine ophthalmic gel 3% required any supplemental treatment (general anesthesia, intraoperative systemic analgesia, or other type) to manage anesthesia throughout the procedure. Further studies are necessary to confirm if pain and patient comfort can be managed with chloroprocaine ophthalmic gel 3% alone during routine cataract surgery.

Safety data confirmed the very favorable safety profile of chloroprocaine 3% gel. Specifically, the proportion of TEAEs was higher in the tetracaine 0.5% eye drop (11.0%) than in the chloroprocaine 3% gel group (8.4%). Importantly, none of the TEAEs recorded in chloroprocaine ophthalmic gel group was considered as related to the study treatment.

Ocular symptoms, slit-lamp examination, corneal fluorescein staining, fundus ophthalmoscopy, IOP, and visual acuity results reported for chloroprocaine ophthalmic gel did not give rise to any local tolerance concern during the conduction of clinical study.

Of note, there were no secondary infections after surgery in the operated eyes, despite the suspected potential barrier effect of gels on povidone-iodine, which has occasionally been reported with other lidocaine gel formulations.^28^ Considering that chloroprocaine 3% gel formulation has a lower viscosity with respect to the other ophthalmic gels on the market, it can be postulated that the viscosity of chloroprocaine 3% does not constitute an antiseptic barrier.

In addition, chloroprocaine 3% ophthalmic gel is provided in a single use container suitable for ocular application, further mitigating potential contamination and secondary infection risks.

## Conclusion

Data collected in the present study demonstrated that, in cataract surgery, chloroprocaine 3% gel is safe, well tolerated, efficacious, and with suitable characteristics in terms of onset and duration. Both investigators and patients were very satisfied with the novel ophthalmic anesthetic, chloroprocaine 3% gel.

The results of this study contributed to the registration of the product in the United States, which is indicated for ocular surface anesthesia. Therefore, Chloroprocaine ophthalmic gel 3% represents a valid therapeutic alternative not only in cataract surgery but also in less invasive ophthalmic procedures.

## References

[1] Bellucci R, Bellucci F. Comparative efficacy of topical tetracaine solution versus lidocaine gel in cataract surgery. Open Access Surg 2012;5:1–8.

[2] Malik A. Efficacy and performance of various local anaesthesia modalities for cataract surgery. J Clin Exp Ophthalmol 2013;S1:007.

[3] Cyriac IC, Pineda R, 2nd. Postoperative complications of periocular anesthesia. Int Ophthalmol Clin 2000;40(1):85–91. 10.1097/00004397-200001000-00009 10713916

[4] Nouvellon E, Cuvillon P, Ripart J, et al. Anaesthesia for cataract surgery. Drugs Aging 2010;27(1):21–38. 20030430 10.2165/11318590-000000000-00000

[5] Page MA, Fraunfelder FW. Safety, efficacy, and patient acceptability of lidocaine hydrochloride ophthalmic gel as a topical ocular anesthetic for use in ophthalmic procedures. Clin Ophthalmol 2009;3:601–609. 19898665 10.2147/opth.s4935 PMC2773282

[6] Busbee BG, Alam A, Reichel E. Lidocaine hydrochloride gel for ocular anesthesia: Results of a prospective, randomized study. Ophthalmic Surg Lasers Imaging 2008;39(5):386–390. 18831420 10.3928/15428877-20080901-03

[7] Shah H, Reichel E, Busbee B. A novel lidocaine hydrochloride ophthalmic gel for topical ocular anesthesia. Local Reg Anesth 2010;3:57–63. 22915870 10.2147/lra.s6453 PMC3417949

[8] Barequet IS, Soriano ES, Green WR, et al. Provision of anesthesia with single application of lidocaine 2% gel. J Cataract Refract Surg 1999;25(5):626–631. 10330634 10.1016/s0886-3350(99)00004-8

[9] Bardocci A, Lofoco G, Perdicaro S, et al. Lidocaine 2% gel versus lidocaine 4% unpreserved drops for topical anesthesia in cataract surgery: A randomized controlled trial. Ophthalmology 2003;110(1):144–149. 12511360 10.1016/s0161-6420(02)01562-2

[10] Soliman MM, Macky TA, Samir MK. Comparative clinical trial of topical anesthetic agents in cataract surgery: Lidocaine 2% gel, bupivacaine 0.5% drops, and benoxinate 0.4% drops. J Cataract Refract Surg 2004;30(8):1716–1720. 15313296 10.1016/j.jcrs.2003.12.034

[11] Covino BG. Pharmacology of LA agents. Br J Anaesth 1986;58:701–716. 2425835 10.1093/bja/58.7.701

[12] McGee HT, Fraunfelder FW. Toxicities of topical ophthalmic anesthetics. Exp Opin Drug Saf 2007;6(6):637–640. 10.1517/14740338.6.6.637 17967152

[13] Chow S, Shao J, Wang H. Sample Size Calculations in Clinical Research. In: Chapman & Hall, ed. CRC Biostatistics Series. 64. 2nd eds. Boca Raton; 2008; pp. 1307–1308.

[14] Irle S, Lückefahr MH, Tho Seeth T. Tetracaine drops versus lidocaine gel for topical anaesthesia in cataract surgery. Klin Monbl Augenheilkd 2003;220(9):625–628. 14533061 10.1055/s-2003-42811

[15] Amiel H, Koch PS. Tetracaine hydrochloride 0.5% versus lidocaine 2% jelly as a topical anesthetic agent in cataract surgery: Comparative clinical trial. J Cataract Refract Surg 2007;33(1):98–100. 17189801 10.1016/j.jcrs.2006.09.013

[16] Chalam KV, Murthy R, Agarwal S, et al. Comparative efficacy of topical tetraVisc versus lidocaine gel in cataract surgery. Biomed Central Ophthalmol 2009;9:7. 10.1186/1471-2415-9-7 PMC2736919 19686592

[17] Tsoumani AT, Asproudis IC, Damigos D. Tetracaine 0.5% eyedrops with or without lidocaine 2% gel in topical anesthesia for cataract surgery. Clin Ophthalmol 2010;4:967–970. 20856590 10.2147/opth.s11755 PMC2938275

[18] Goodman and Gilman's. The Pharmacological Basis of Therapeutics, 12th Ed. New York: MacGraw-Hill. Chapter. 14 Las; 2011.

[19] Covino BG, Giddon DB. Pharmacology of local anesthetic agents. J Dent Res 1981;60:1454–1459. 6942010 10.1177/00220345810600080903

[20] Gan L, Wang J, Jiang M, et al. Recent advances in topical ophthalmic drug delivery with lipid-based nanocarriers. Drug Discov Today 2013;18:290–297. 23092895 10.1016/j.drudis.2012.10.005

[21] Resnikoff S, Pascolini D, Etya'ale D, et al. Global data on visual impairment in the year 2002. Bull World Health Organ 2004;82(11):844–851. 15640920 PMC2623053

[22] Prokofyeva E, Wegener A, Zrenner E. Cataract prevalence and prevention in Europe: A literature review. Acta Ophthalmol 2013;91(5):395–405. 22715900 10.1111/j.1755-3768.2012.02444.x

[23] Jiang L, Zhang K, He W, et al. Perceived pain during cataract surgery with topical anesthesia: A comparison between first-eye and second-eye surgery. J Ophthalmol 2015;2015:383456. 26064671 10.1155/2015/383456 PMC4434194

[24] Donnenfeld ED, Shojaei RD. Effect of intracameral phenylephrine and ketorolac 1.0%/0.3% on intraoperative pain and opioid use during cataract surgery. Clin Ophthalmol 2019;13:2143–2150. 31806927 10.2147/OPTH.S229515 PMC6839573

[25] Russell KM, Warner ME, Erie JC, et al. Anesthesia recovery after ophthalmologic surgery at an ambulatory surgical center. J Cataract Refract Surg 2019;45(6):823–829. 31146933 10.1016/j.jcrs.2019.01.017

[26] Jackson KJ, Kim T. Prevalence of opioid usage with monitored anesthesia care in cataract surgery: A single-site review. J Cataract Refract Surg 2023;49(9)996–997. 37639310 10.1097/j.jcrs.0000000000001238

[27] Zakzewski P, Friel T, et al. Monitored anesthesia care provided by registered respiratory care practitioners during cataract surgery: A report of 1957 cases. Ophthalmology 2005;112(2)272–277. 15691563 10.1016/j.ophtha.2004.08.016

[28] Boden JH, Myers ML, Lee T, et al. Effect of lidocaine gel on povidone-iodine antisepsis and microbial survival. J Cataract Refract Surg 2008;34(10):1773–1775.1. 18812132 10.1016/j.jcrs.2008.05.056

